# Genus *Microsternus* Lewis (1887) from China, with description of a new genus *Neosternus* from Asia (Coleoptera, Erotylidae, Dacnini)

**DOI:** 10.3897/zookeys.340.6044

**Published:** 2013-10-04

**Authors:** Cong-Chao Dai, Mei-Jun Zhao

**Affiliations:** 1 Department of Biology, College of Life and Environmental Sciences, Shanghai Normal University, Shanghai, 200234, P. R. China

**Keywords:** Coleoptera, Erotylidae, Dacnini, *Microsternus*, *Neosternus*, identification key, new Genus, new species, new synonym, Asia, China

## Abstract

This work treats species of the genus *Microsternus* Lewis, 1887 from Asia and North America. A new genus is described: *Neosternus* (type species *Microsternus higonius* Lewis, 1887). A new species is described: *Microsternus pengzhongi*. A new synonym is provided: *Microsternus tricolor taiwanicus* Nakane (=*Microsternus tricolor* Lewis). Three species previously placed in *Microsternus* Lewis, 1887 are transferred to *Neosternus* resulting in the following three new combinations: *Neosternus higonius* (Lewis, 1887), *Neosternus taiwanus* (Chûjô, 1976), and *Neosternus hisamatsui* (Nakane, 1981).

## Introduction

### General introduction

The family Erotylidae is composed of fungus feeding beetles which vary greatly in body size and color; many are elaborately patterned. Crotch’s (1876) world revision of the Erotylidae was the last study to cover the entire Old World fauna at the species level, but it provided no keys nor illustrations, and only a few short descriptions. In the time between Chûjô and Chûjô’s (1988) catalog of the Old World erotylids and Wegrzynowicz’s (2006) catalog of the Palaearctic erotylids, the China erotylid fauna had been studied intermittently, with scattered regional studies, checklists, and species descriptions. Most Chinese erotylid genera have no modern revisions.

### Taxonomic arrangement

Wegrzynowicz (2002) formally synonymized the tribes Megalodacnini and Encaustini. More recent phylogenetic work by Leschen (2003), Robertson et al. (2004), and Leschen and Buckley (2007) were either inconclusive or resulted in different tree topologies that indicate more work is needed to better establish relationships of all tribes before making further taxonomic changes.

Herein, tribal placement of included taxa follows a five-tribe system: Dacnini, Megalodacnini, Encaustini, Erotylini, Tritomini (Lawrence and Newton 1995). China has three genera arranged under tribe the Dacnini: *Dacne*, *Microsternus* and *Neosternus* gen. n.

## History

The first described member of the genus *Microsternus* Lewis is *Megalodacne ulkei* Crotch (1873). In studying the Japanese erotylid specimens, Lewis (1883) described *Episcapha perforata*, but on further study of these group, Lewis (1887a) found three similar Japanese species required a genus to be formed for their reception. Together with *Episcapha perforata*, Lewis thought these four species were congeneric with American species *Megalodacne ulkei* and established the genus *Microsternus*. Lewis (1887b) transferred *Episcapha perforata* into *Microsternus* becoming *Microsternus perforatus* (Lewis, 1883) and described three other species as *Microsternus higonius* Lewis (1887b), *Microsternus crotchi* Lewis (1887b) and *Microsternus tricolor* Lewis (1887b).

Before our study, there were 17 valid species and 1 subspecies placed in the genus *Microsternus* Lewis: *Microsternus ulkei* Crotch (1873), *Microsternus perforatus* (Lewis, 1883), *Microsternus crotchi* Lewis (1887b), *Microsternus higonius* Lewis (1887b), *Microsternus tricolor* Lewis (1887b), *Microsternus cribricollis* Gorham (1895), *Microsternus puncticollis* Heller (1918), *Microsternus queenslandicus* Heller (1918), *Microsternus javanus* Deelder (1942), *Microsternus sumatranus* Deelder (1942), *Microsternus tokioensis* Nakane (1961), *Microsternus yamadai* Chûjô & Shibata (1963), *Microsternus tricolor taiwanicus* Nakane (1966), *Microsternus quatei* Chûjô (1968), *Microsternus bhutanensis* Chûjô (1975), *Microsternus taiwanus* Chûjô (1976), *Microsternus hisamatsui* Nakane (1982), *Microsternus nakanoi* Narukawa (2004). Only one species and one subspecies have been reported from China (Taiwan), *Microsternus taiwanus* and *Microsternus tricolor taiwanicus*. No record has been reported from the Chinese mainland.

In this work, while examining the specimens of *Microsternus* from China, we described a new species *Microsternus pengzhongi* sp. n. from Hainan Prov. and reduce *Microsternus tricolor taiwanicus* Nakane to a synonym of *Microsternus tricolor* Lewis. After genitalic dissections and comparisons of head and pronotum structures, we recognized that the genus *Microsternus* can be divided in two species-groups. *Microsternus higonius* Lewis (1887b), *Microsternus taiwanus* Chûjô (1976) and *Microsternus hisamatsui* Nakane (1982) are not congeneric with the type species of *Microsternus* Lewis, *Microsternus ulkei* Crotch (1873). Unlike typical *Microsternus*, the species, *Microsternus higonius*, *Microsternus taiwanus* and *Microsternus hisamatsui* do not have the pit posterior to the postmandibular lobes; pronotum has a deep sulcus along each side, which is broadly margined and formed thicken lines in lateral view; male genitalia with flagellum curved, bearing a dorsal, arched, cartilaginous mass on apical quarter, along with prosternum and mesosternum characters not present in other members of Dacnini. Here we describe a new genus *Neosternus* for these three species.

## Characters and terminology

### Male genitalia

The internal sac of the male genitalia is held invaginated within the median lobe. During copulation, the internal sac is everted; exposing any microstructure and extending the flagellum. The median lobe, internal sac, and flagellum are the true copulatory organs and show the majority of species specific characters. For additional insight into the genitalia of Erotylidae see Skelley (1998) and Skelley and Leschen (2007).

In this work, after dissection of the internal sac, we found the dorsal lobe of *Microsternus* has obvious differences that are important characters in species recognition.

### Female genitalia

Female genitaliaseemed to vary little from species to species. Structures varied in proportions, but species recognition based on female genitalia was not possible with any degree of confidence. The sclerotized spermatheca showed some variation in shape which could be useful to determine relationships within the genus. See Skelley (1998).

## Material and methods

In addition to extensive collecting by the authors and their colleagues, Chinese specimens of the tribe Dacnini were borrowed from Wen-xuan Bi’s private collections. North America specimens of the genus *Microsternus* were provided for study from Florida State Collection of Arthropods, USA [Paul E. Skelley]. The photos of type specimens were taken from The Natural History Museum, London, England.

Erotylids were collected in a wide variety of woodland fungi, in crevices under bark or in other retreats by splitting and sifting, and in light traps. For an examination of the male genitalia, the abdominal segments were detached from the body after softening in hot water. The genitalia, together with other dissected parts, were mounted in Euparal (Chroma Gesellschaft Schmidt, Koengen, Germany) on plastic slides. Photos of sexual characters were taken with a FUJIFILM X10 camera attached to an Olympus SZX 16 stereoscope; habitus photos were taken with a Canon macro photo lens MP-E 65 mm attached to a Canon EOS7D camera.

The specimens treated in this study are deposited in the following public and private collections:

SNUC Department of Biology, Shanghai Normal University, P. R. China

FSCA Florida State Collection of Arthropods, USA [Paul E. Skelley]

NHM The Natural History Museum, London, England

CBWX CollectionofWen-xuan, Bi, Shanghai, China

## Taxonomy

### Key to genus of Dacnini from China

**Table:** 

1	Mesosternum exposed; eyes not very coarsely facetted	*Dacne* Latreille
–	Prosternal process covering the mesosternum; eyes very coarsely facetted	2
2	Head with the pit posterior to the postmandibular lobes, pronotum without a deep sulcus along each side and narrowly margined in lateral view	*Microsternus* Lewis
–	Head without the pit posterior to the postmandibular lobes, pronotum with a deep sulcus along each side, which is broadly margined in lateral view	*Neosternus* Dai & Zhao, gen. n.

### Genus Microsternus

Lewis, 1887

http://species-id.net/wiki/Microsternus

Figs 1–2 , 3–6 , 7–8 , 9–12 , 13–14 , 15–21 , 22–23 , 24–29 , 30–33 , 40–42 , 45–47 , 50–52 , 59–64 , 73–74 , 77–78

#### Type species.

*Megalodacne ulkei* Crotch, 1873

#### Description.

**Figures 1–2.**
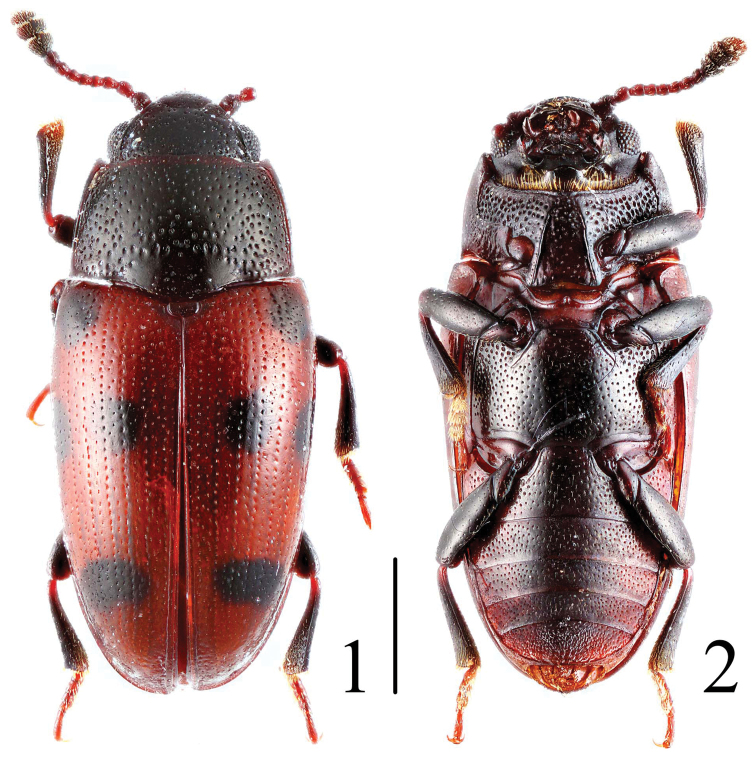
Habitus of *Microsternus ulkei* in dorsal and ventral view. Scale = 1 mm.

Body (Figs 1–2, 7–8, 13–14, 22–23, 73–74, 77–78) small, elongate, with legs short and robust, the tarsi cylindrical, 5-jointed, the four basal joints short, nearly equal in size and not at all dilated, the last joint long. Antennae not very long, the latter with a broad 3-jointed club. Eyes coarsely facetted. Head with the pit posterior to the postmandibular lobes (Figs 40–42). Maxillary and labial terminal palpomeres acuminate, sensory area restricted to apex. Pronotum (Figs 45–47) arched, widest at base; narrowed from base to apex, with formed thinned lines in lateral view (Figs 50–52); disk punctured, except the impunctate medio-basal area, which is limited by an arched transverse row of coarse punctures. Prosternum with median area including its process elevated in an elongate triangular plane, which is distinctly bordered by a ridge on both sides and shortly rounded-subtruncate in front. Mesosternum almost concealed by prosternal process. Metasternum with a pair of mesocoxal lines strongly divergent posteriorly. Abdomen without metacoxal lines on basal visible sternite. Elytra convex.

**Figures 3–6.**
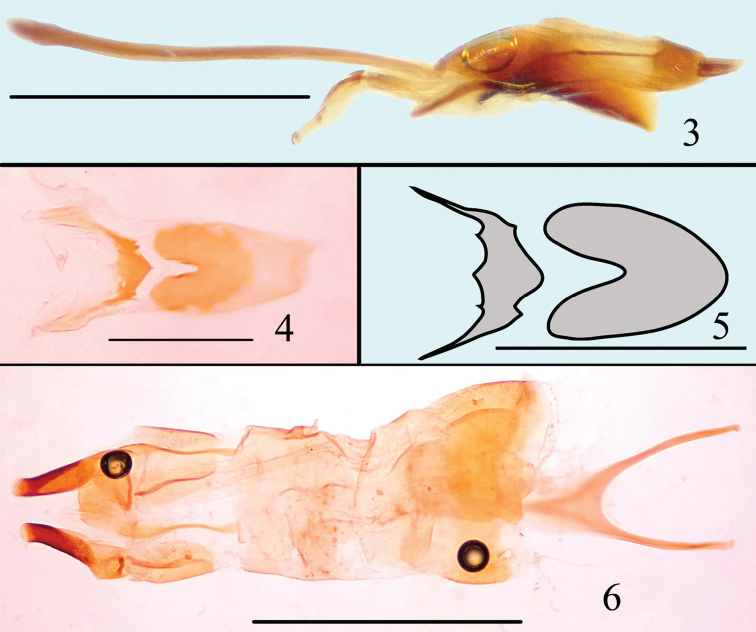
Male genitalia of *Microsternus ulkei* in lateral view **3** Dorsal lobe of *Microsternus ulkei* in dorsal view **4–5** Female genitalia of *Microsternus ulkei* in dorsal view **6**. Scale = 1mm (**3, 6**), Scale = 0.2mm (**4**, **5**).

**Figures 7–8.**
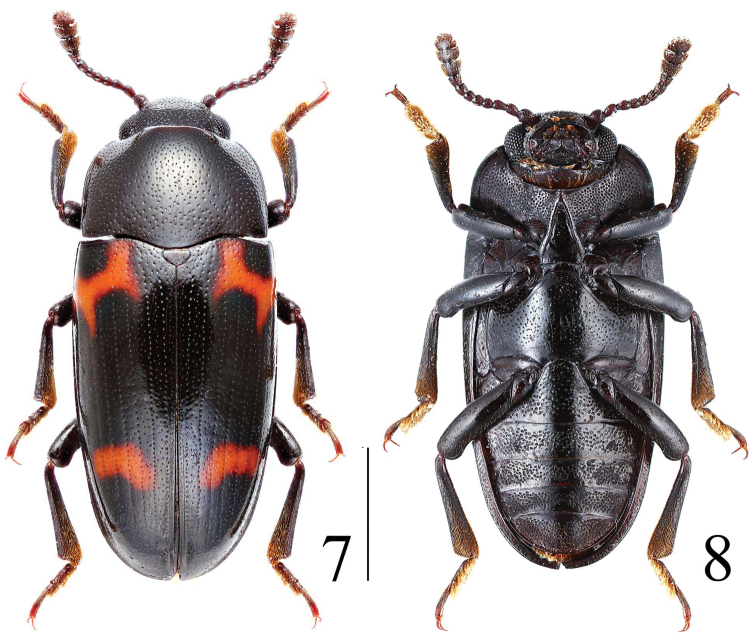
Habitus of *Microsternus pengzhongi* in dorsal and ventral view. Scale = 2 mm.

**Figures 9–12.**
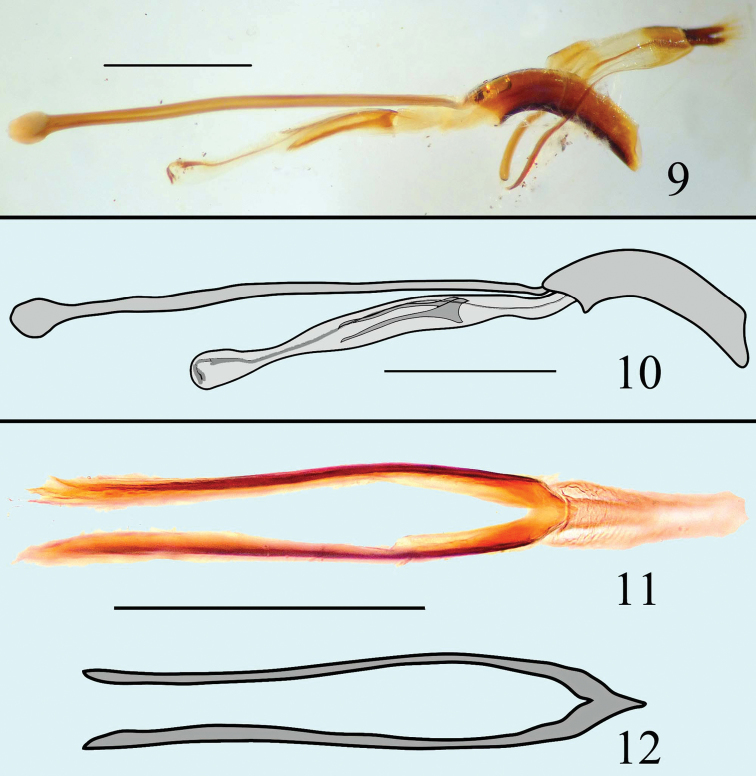
Male genitalia of *Microsternus pengzhongi* in lateral view **9–10** Dorsal lobe of *Microsternus pengzhongi* in dorsal view **11–12**. Scale=1mm (**9**, **10**), Scale=0.5mm (**11**, **12**).

**Figures 13–14.**
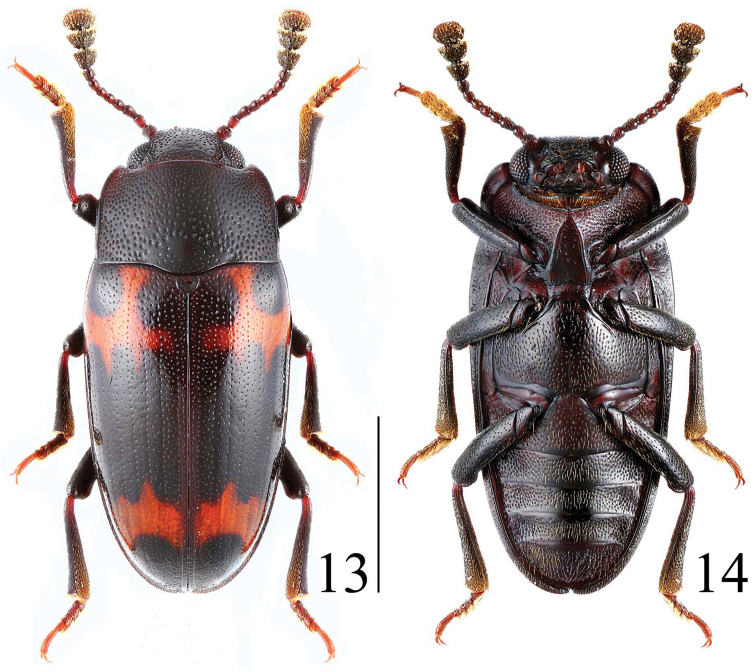
Habitus of *Microsternus perforatus* in dorsal and ventral view. Scale = 2 mm.

**Figures 15–21.**
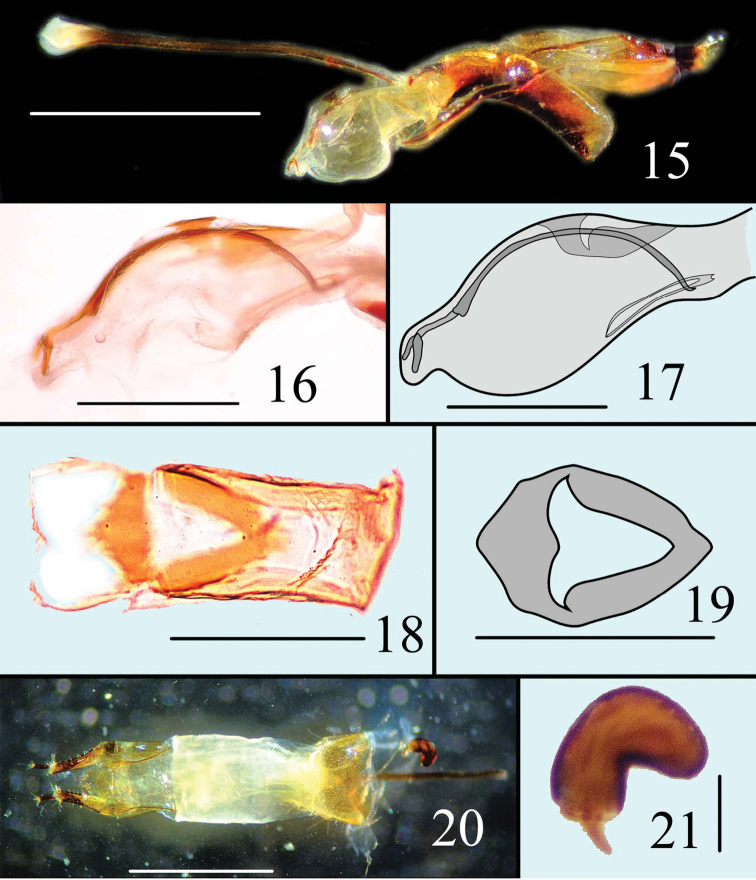
Male genitalia of *Microsternus perforatus* in lateral view **15** Internal sac of *Microsternus perforatus* in lateral view **16–17** Dorsal lobe of *Microsternus perforatus* in dorsal view **18–19** Female genitalia of *Microsternus perforatus* in dorsal view **20** Female spermatheca of *Microsternus perforatus* **21**. Scale=1mm (**15**, **20**), Scale=0.3mm (**16**, **17**), Scale=0.2mm (**18**, **19**), Scale=0.1mm (**21**).

**Figures 22–23.**
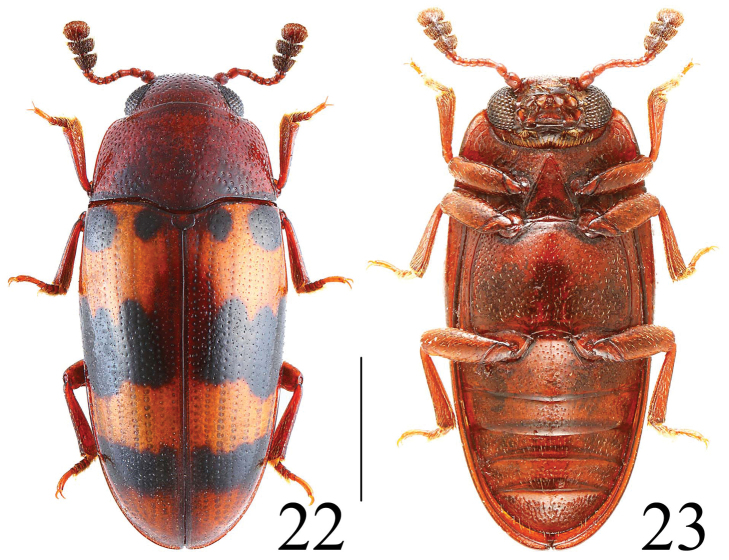
Habitus of *Microsternus tricolor* in dorsal and ventral view. Scale = 1 mm.

**Figures 24–29.**
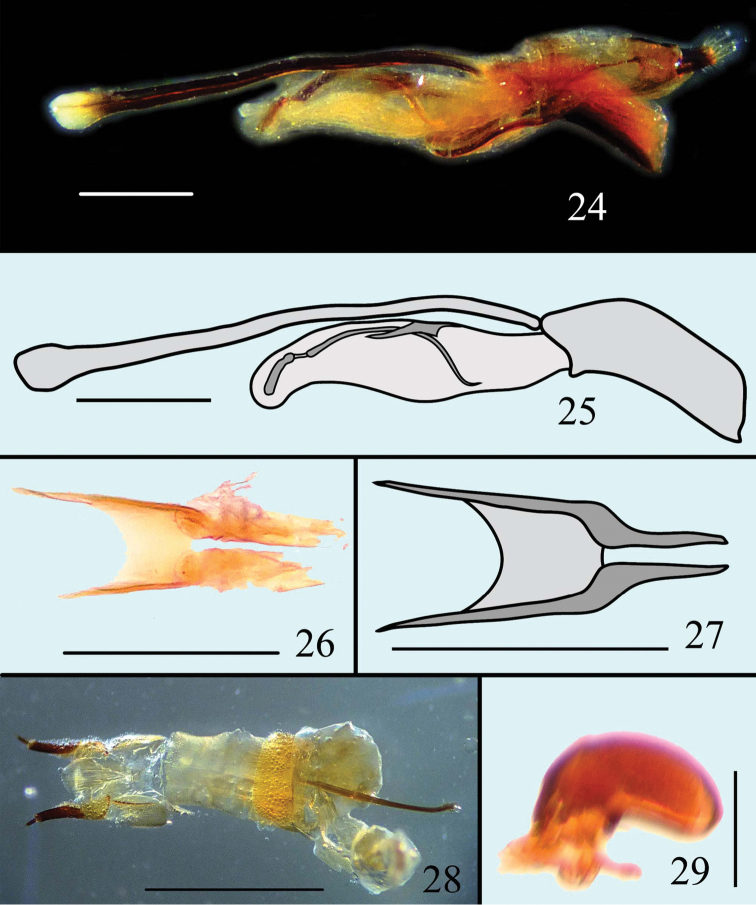
Male genitalia of *Microsternus tricolor* in lateral view **24–25** Dorsal lobe of *Microsternus tricolor* in dorsal view **26–27** Female genitalia of *Microsternus tricolor* in dorsal view **28** Female spermatheca of *Microsternus tricolor* **29**. Scale=1mm (**28**), Scale=0.3mm (**24**, **25**), Scale=0.2mm (**26**, **27**), Scale=0.1mm (**29**).

**Figures 30–33.**
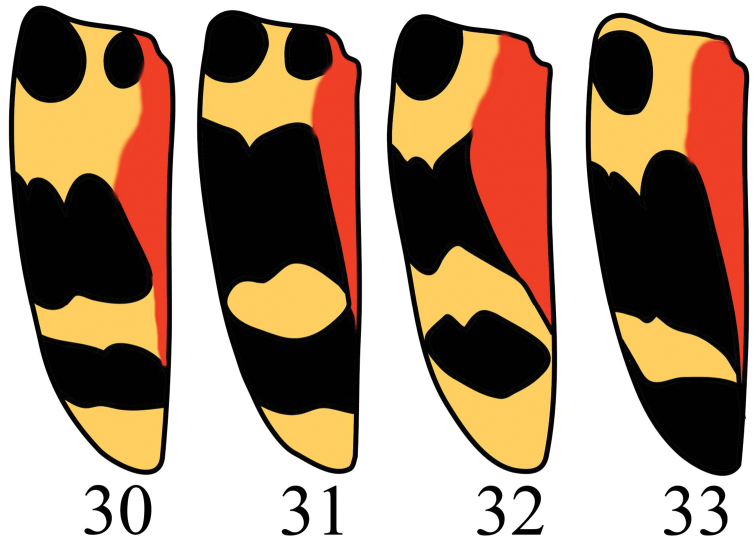
Elytra of *Microsternus tricolor* in dorsal view.

#### Distribution.

*Microsternus* is widespread in Asia, with one species in Australia and one species in America.

#### Diagnosis.

Defining characters for *Microsternus* are body elongate, head with the pit posterior to the postmandibular lobes, pronotum without a deep sulcus along each side and narrowed margin in lateral view, mesosternum almost concealed by prosternal process, male genitalia with flagellum (Figs 59–64) bearing a straight mass on apical quarter.

#### Key to Chinese species of genus *Microsternus*

**Table:** 

1	Body black to reddish-brown, each elytra black with two narrow orange bands	2
–	Body red, elytra with orange and black spots or bands (Figs 22–23)	*Microsternus tricolor* (Lewis)
2	Body shining, punctation of pronotum fine and sparse (Figs 7–8)	*Microsternus pengzhongi* Dai & Zhao, sp. n.
–	Body not shining, punctation of pronotum coarse and close (Figs 13–14)	*Microsternus perforatus* (Lewis)

### Microsternus pengzhongi

Dai & Zhao sp. n.

http://zoobank.org/87B144AA-4704-4A78-8A8E-0ACABC75B973

http://species-id.net/wiki/Microsternus_pengzhongi

Figs 7–8 , 9–12

#### Material examined.

**Holotype: CHINA: Hainan Prov.:** ♂, Jianfengling N.R., Mingfenggu Valley, 18°44'N, 108°50'E, alt. 950 m, 30.IV.2012, Peng & Dai leg. (SNUC).

#### Description.

Body (Fig. 7, 8) elongate, length: 7.5 mm; width: 3.2 mm. Body black, shining. Each elytron with two narrow orange bands.

Head width between eyes = 4 times eye diameter in dorsal view; punctation fine, spare, separated by two to four puncture diameters; epistome truncate, lacking marginal line on anterior margin; stridulatory files not evident. Eyes coarsely facetted. Antennomere III about 1.2 times as long as IV; antennomere VIII slightly wider than VII, about 1.3 times as wide as long; antennomere IX trapezoidal; antennomere X transverse; antennomere XI almost elliptic. Gular area with pit posterior to the postmandibular lobes. Maxillary and labial terminal palpomeres acuminate, sensory area restricted to apex. Mentum broad with anterior projection, almost triangular, slightly more than 2.3 times wider than long.

Pronotum arched, widest at base (pl/pw = 0.65); narrowed from base to apex, with formed thinned lines in lateral view; disk finely and spare punctured, except the impunctate medio-basal area, which is limited by an arched transverse row of fine punctures.

Prosternum with median area including its process elevated in an elongate triangular plane, which is distinctly bordered by a ridge on both sides and shortly rounded-subtruncate in front, bearing a few fine punctures; sides rugose, coarsely and densely punctured. Mesosternum almost conceled by prosternal process, impunctate as the mesepisterna, which is somewhat concave. Metasternum rather sparsely and strongly punctured on lateral areas, some finer punctures on median area, with a pair of mesocoxal lines strongly divergent posteriorly. Abdomen rather strongly and closely punctured, but median areas of four basal visible sternites and medio-basal area of last visible sternite with few punctures respectively; without metacoxal lines on basal visible sternite. Legs rather robust.

Scutellum pentagonal, finely and sparely punctured.

Elytra convex, with eight striae of distinct punctures on each elytron and each interstice with a row of extremely fine punctures.

Male genitalia (Figs 9–10) with flagellum bearing a straight mass on apical; flagellar apex acute with a well-separated ventral process; dorsal lobe of internal sac with long and tweezer-like structure in dorsal view (Figs 11–12).

#### Distribution.

China (Hainan Province).

#### Diagnosis.

Characterized by its shining body, spare punctured pronotum and dorsal lobe’s unique structure of internal sac.

#### Etymology.

This species is named in honor of Mr. Zhong Peng, one of the collectors of this new species.

### Microsternus perforatus

Lewis, 1883

http://species-id.net/wiki/Microsternus_perforatus

Figs 13–14 , 15–21 , 42 , 47 , 52 , 63–64 , 74–75 , 78

#### Material examined.

**CHINA: Hainan Prov.:** 4♂♂, 3♀♀, Shuiman County, Mt. Wuzhishan, 18°54'N, 109°41'E, alt. 500–800 m, 24.IV.2011, Bi Wenxuan leg. (CBWX); **Zhejiang Prov.:** 1♂♂, 2♀♀, Linan County, Mt. Tianmushan, 18°54'N, 109°41'E, alt. 300 m, 27.IV.2008, He & Tang leg. (SNUC)

#### Description.

Body (Figs 13–14, 74–75, 78) elongate, length: 4.9–7.0 mm; width: 2.2–3.0 mm. Body black to blackish-brown. Each elytron with two narrow orange bands.

Head width between eyes = 7 times eye diameter in dorsal view; punctation coarse, close, separated by half to two puncture diameters; epistome truncate, lacking marginal line on anterior margin; stridulatory files not evident. Eyes coarsely facetted. Antennomere III about 1.4 times as long as IV; antennomere VIII slightly wider than VII, about 1.2 times as wide as long; antennomere IX trapezoidal; antennomere X transverse; antennomere XI almost elliptic. Gular area with pit posterior to the postmandibular lobes (Fig. 42). Maxillary and labial terminal palpomeres acuminate, sensory area restricted to apex. Mentum broad with anterior projection, almost triangular, slightly more than 3.7 times wider than long.

Pronotum (Fig. 47) arched, widest at base (pl/pw = 0.63); narrowed from base to apex, with formed thinned lines in lateral view (Fig. 52); disk coarsely and close punctured, except the impunctate medio-basal area, which is limited by an arched transverse row of coarse punctures.

Prosternum with median area including its process elevated in an elongate triangular plane, which is distinctly bordered by a ridge on both sides and shortly rounded-subtruncate in front, bearing a few fine punctures; sides rugose, coarsely and densely punctured. Mesosternum almost conceled by prosternal process, impunctate as the mesepisterna, which is somewhat concave. Metasternum rather sparsely and strongly punctured on lateral areas, some finer punctures on median area, with a pair of mesocoxal lines strongly divergent posteriorly. Abdomen rather strongly and closely punctured, but median areas of four basal visible sternites and medio-basal area of last visible sternite with few punctures respectively; without metacoxal lines on basal visible sternite. Legs rather robust.

Scutellum pentagonal, with each corner rounded, flattish and almost impunctate on surface.

Elytra convex, with eight striae of distinct punctures on each elytron and each interstice with a row of extremely fine punctures.

Male genitalia (Fig. 15–17) with flagellum bearing a straight mass on apical quarter; flagellar (Fig. 63–64) apex acute with a well-separated ventral process; dorsal lobe of internal sac with subhexagon edge and propeller -like hollow in dorsal view (Figs 18–19).

Female genitalia (Fig. 20) and spermatheca (Fig. 21) simple.

#### Distribution.

China, Japan.

#### Diagnosis.

Characterized by its close punctured pronotum, orange bands of elytra and dorsal lobe’s unique structure of internal sac.

### Microsternus tricolor

Lewis, 1887

http://species-id.net/wiki/Microsternus_tricolor

Figs 22–23 , 24–29 , 30–33 , 41 , 46 , 51 , 61–62 , 73 , 77

Microsternus tricolor taiwanicus Nakane, 1966, syn. n.

#### Material examined.

**CHINA: Hainan Prov.:** 1♂♂, 1♀, Shangsi County, Mt. Wuzhishan, 18°54'N, 109°41'E, alt. 800–1000 m, 22.IV.2011, Bi Wenxuan leg. (CBWX); 1♂, Jianfenglin N R. Mingfenggu Valley, 18°44'N, 108°50'E, alt. 1000 m, 22. V.2011, Bi Wenxuan leg. (CBWX)

#### Description.

Body (Figs 22–23, 73, 77) elongate, length: 3.0–5.0 mm; width: 1.4–2.4 mm. Body red to reddish-brown. Each elytron orange and black spots or bands.

Head width between eyes = 8 times eye diameter in dorsal view; punctation coarse, close, separated by half to two puncture diameters; epistome truncate, lacking marginal line on anterior margin; stridulatory files not evident. Eyes coarsely facetted. Antennomere III about 1.7 times as long as IV; antennomere VIII slightly wider than VII, about 1.2 times as wide as long; antennomere IX trapezoidal; antennomere X transverse; antennomere XI almost elliptic. Gular area with pit posterior to the postmandibular lobes (Fig. 41). Maxillary and labial terminal palpomeres acuminate, sensory area restricted to apex. Mentum broad with anterior projection, almost triangular, slightly more than 4.0 times wider than long.

Pronotum (Fig. 46) arched, widest at base (pl/pw = 0.58); narrowed from base to apex, with formed thinned lines in lateral view (Fig. 51); disk coarsely and close punctured, except the impunctate medio-basal area, which is limited by an arched transverse row of coarse punctures.

Prosternum with median area including its process elevated in an elongate triangular plane, which is distinctly bordered by a ridge on both sides and shortly rounded-subtruncate in front, bearing a few fine punctures; sides rugose, coarsely and densely punctured. Mesosternum almost conceled by prosternal process, impunctate as the mesepisterna, which is somewhat concave. Metasternum rather sparsely and strongly punctured on lateral areas, some finer punctures on median area, with a pair of mesocoxal lines strongly divergent posteriorly. Abdomen rather strongly and closely punctured, but median areas of four basal visible sternites and medio-basal area of last visible sternite with few punctures respectively; without metacoxal lines on basal visible sternite. Legs rather robust.

Scutellum pentagonal, with each corner rounded, flattish and almost impunctate on surface.

Elytra convex, with eight striae of distinct punctures on each elytron and each interstice with a row of extremely fine punctures.

Male genitalia (Figs 24–25) with flagellum bearing a straight mass on apical quarter; flagellar (Figs 61–62) apex acute with a well-separated ventral process; dorsal lobe of internal sac with spade -like structure in dorsal view (Figs 26–27).

Female genitalia (Fig. 28) and spermatheca (Fig. 29) simple.

#### Distribution.

China, Japan, Russia (Far East), Oriental region.

#### Diagnosis.

*Microsternus tricolor* is characterized by its close punctured pronotum, orange and black interphased bands of elytra (Figs 30–33) and dorsal lobe’s unique structure of internal sac.

#### Comment.

Nakane described ‘*taiwanicus*’ for a single female specimen, according his description, *Microsternus tricolor taiwanicus* can be distinguished from *Microsternus tricolor* by a difference in the elytral bands: *Microsternus tricolor taiwanicus* has the median black patch on each side is nearly quadrate, not triangular and more extended inwards, with the inner margin more longitudinal and the front angle nearly rectangular; the posterior yellow fascia is much broader and nearly as wide as the black band before the apex. On studying the specimens of *Microsternus tricolor*, we find the bands of elytra are variable in different specimens (Figs 30–33), after compare the photos about *Microsternus tricolor* from Taiwan and Japan, we think the differences Nakane mentioned in his paper are not unique to Taiwan.

Based on the information outlined above, we considered *Microsternus tricolor taiwanicus* is a new synonym of *Microsternus tricolor*.

### Genus Neosternus

Dai & Zhao gen. n.

http://zoobank.org/1568C347-1E24-4D94-8DAB-4F603588102F

http://species-id.net/wiki/Neosternus

Figs 34–37 , 38–39 , 43–44 , 48–49 , 53–54 , 55–58 , 65–68 , 69–72 , 76

#### Type species.

*Microsternus higonius* Lewis, 1887, here designated.

#### Description.

**Figures 34–37.**
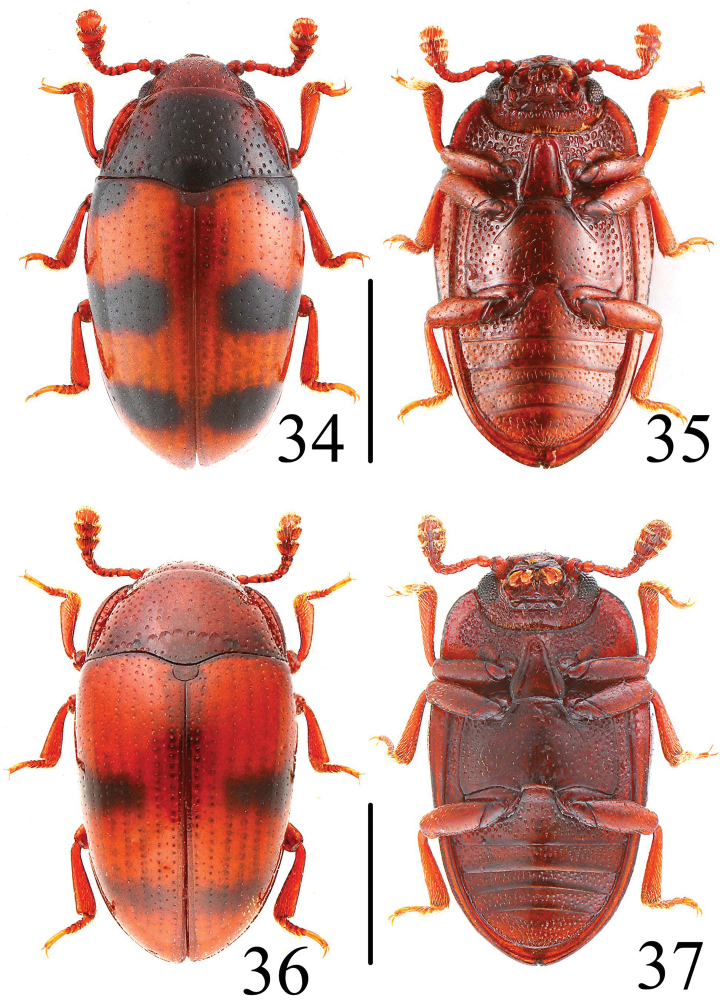
Habitus of *Neosternus higonius* in dorsal and ventral view **34–35** Habitus of *Neosternus hisamatsui* in dorsal and ventral view **36–37**. Scale = 1 mm.

**Figures 38–39.**
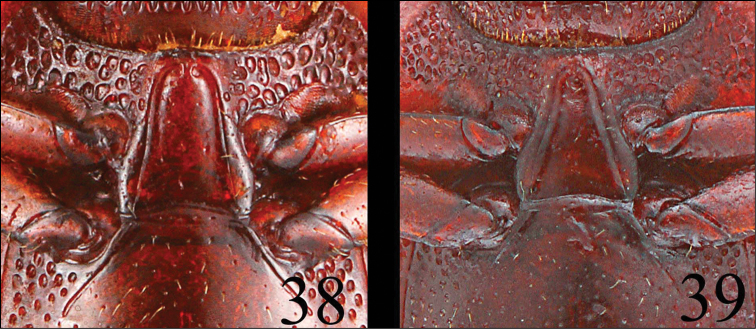
Prosternum of *Neosternus higonius* **38** Prosternum of *Neosternus hisamatsui* **39**.

Body small, elongate oval (Figs 34–37, 76), with legs short and robust, the tarsi cylindrical, 5-jointed, the four basal joints short, nearly equal in size and not at all dilated, the last joint long. Antennae not very long, the latter with a broad 3-jointed club. Eyes coarsely facetted. Maxillary and labial terminal palpomeres (Figs 43, 44) acuminate, sensory area restricted to apex. Pronotum (Figs 48, 49) arched, widest at base; narrowed from base to apex, with a deep sulcus along each side, which is broadly margined and the bordering gradually widened anteriorly, which formed thicken lines in lateral view (Figs 53, 54); disk coarsely and sparsely punctured, except the impunctate medio-basal area, which is limited by an arched transverse row of coarse punctures. Prosternum (Figs 38, 39) with median area including its process elevated in an elongate triangular plane, which is distinctly bordered by a ridge on both sides and shortly rounded-subtruncate in front. Mesosternum almost concealed by prosternal process. Metasternum with a pair of mesocoxal lines strongly divergent posteriorly. Abdomen without metacoxal lines on basal visible sternite. Elytra strongly convex.

**Figures 40–44.**
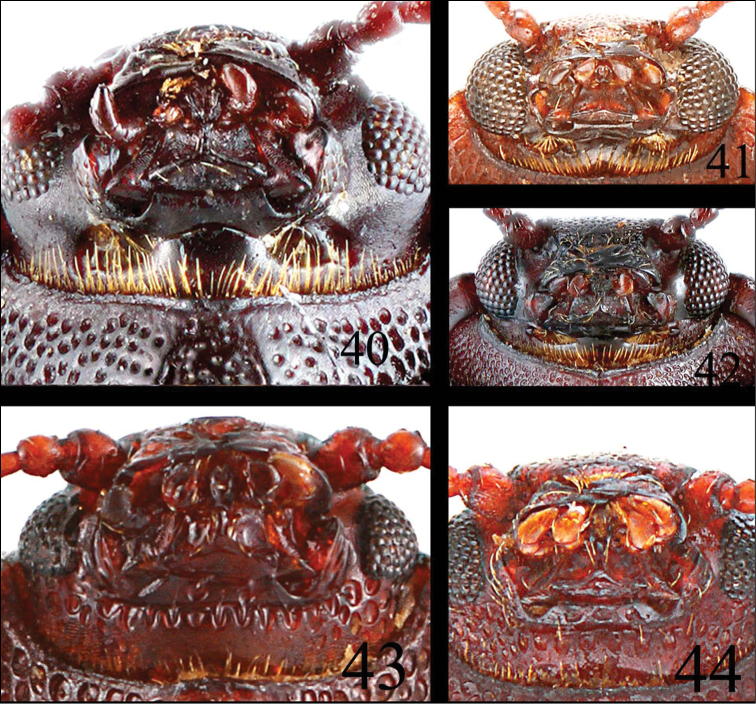
Mouth part of *Microsternus ulkei* **40** Mouth part of *Microsternus tricolor* **41** Mouth part of *Microsternus perforatus* **42** Mouth part of *Neosternus higonius* **43** Mouth part of *Neosternus hisamatsui* **44**.

**Figures 45–49.**
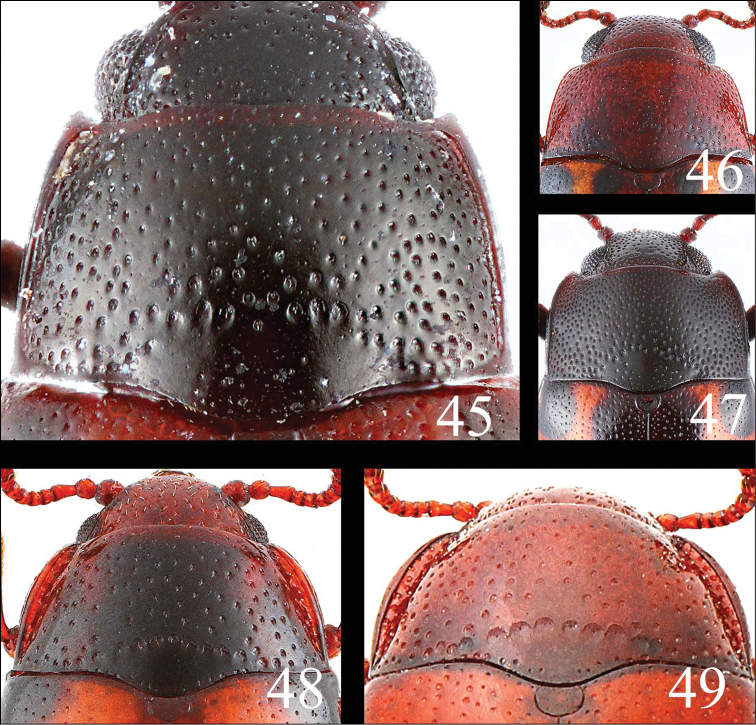
Pronotum of *Microsternus ulkei* in dorsal view **45** Pronotum of *Microsternus tricolor* in dorsal view **46** Pronotum of *Microsternus perforatus* in dorsal view **47** Pronotum of *Neosternus higonius* in dorsal view **48** Pronotum of *Neosternus hisamatsui* in dorsal view **49**.

**Figures 50–54.**
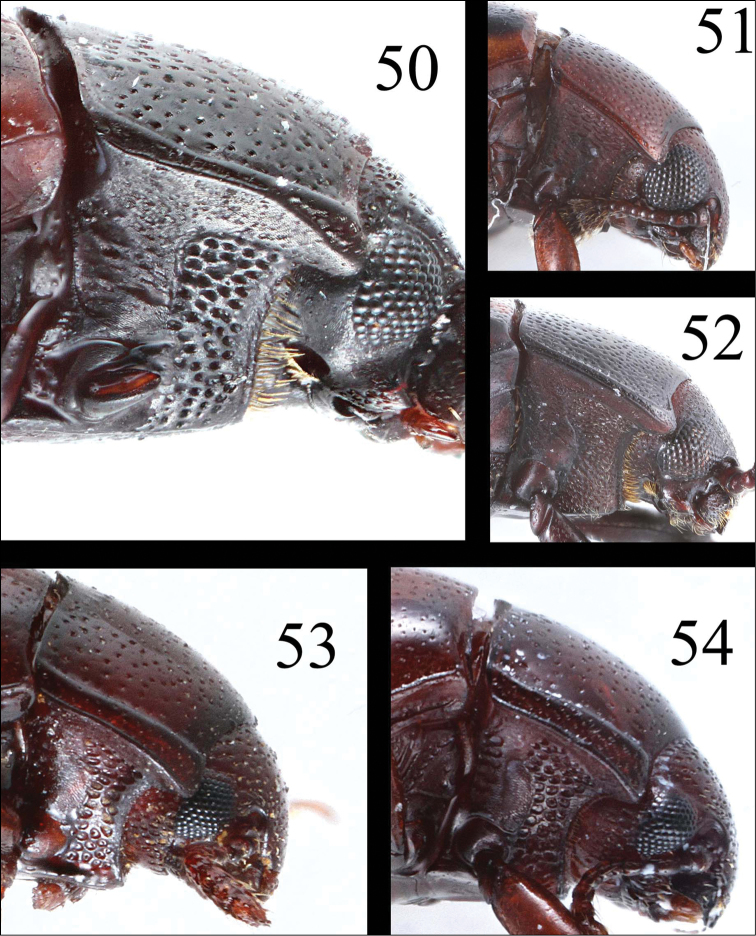
Pronotum of *Microsternus ulkei* in lateral view **50** Pronotum of *Microsternus tricolor* in lateral view **51** Pronotum of *Microsternus perforatus* in lateral view **52** Pronotum of *Neosternus higonius* in lateral view **53** Pronotum of *Neosternus hisamatsui* in lateral view **54**.

**Figures 55–64.**
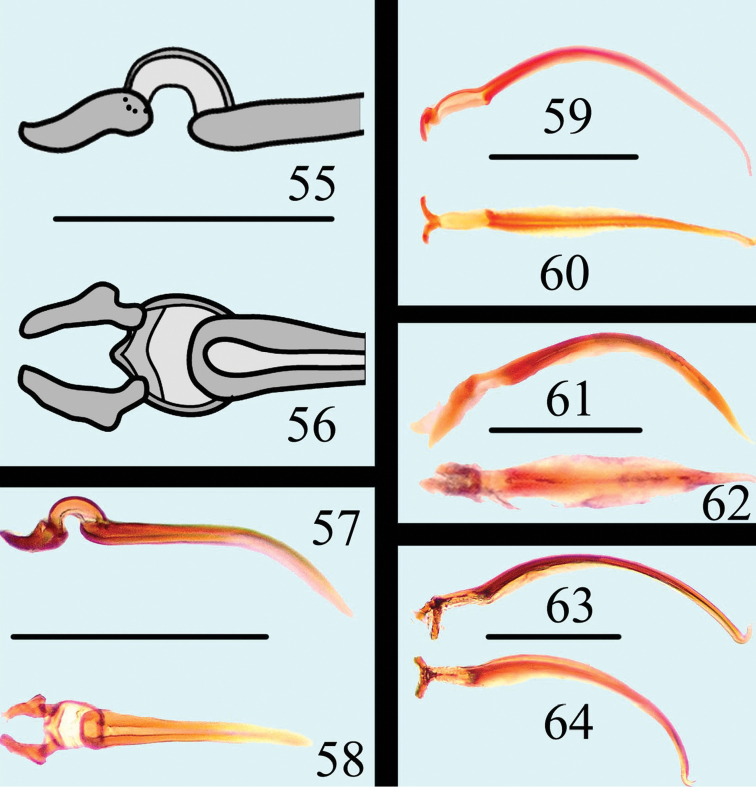
Flagellum of *Neosternus higonius* in lateral view **55** Flagellum of *Neosternus higonius* in dorsal view **56** Flagellum of *Neosternus hisamatsui* in lateral view **57** Flagellum of *Neosternus hisamatsui* in dorsal view **58** Flagellum of *Microsternus ulkei* in lateral view **59** Flagellum of *Microsternus ulkei* in dorsal view **60** Flagellum of *Microsternus tricolor* in lateral view **61** Flagellum of *Microsternus tricolor* in dorsal view **62** Flagellum of *Microsternus perforatus* in lateral view **63** Flagellum of *Microsternus perforatus* in dorsal view **64**. Scale=0.1mm (**55–56**), Scale=0.2mm (**57–64**).

**Figures 65–68.**
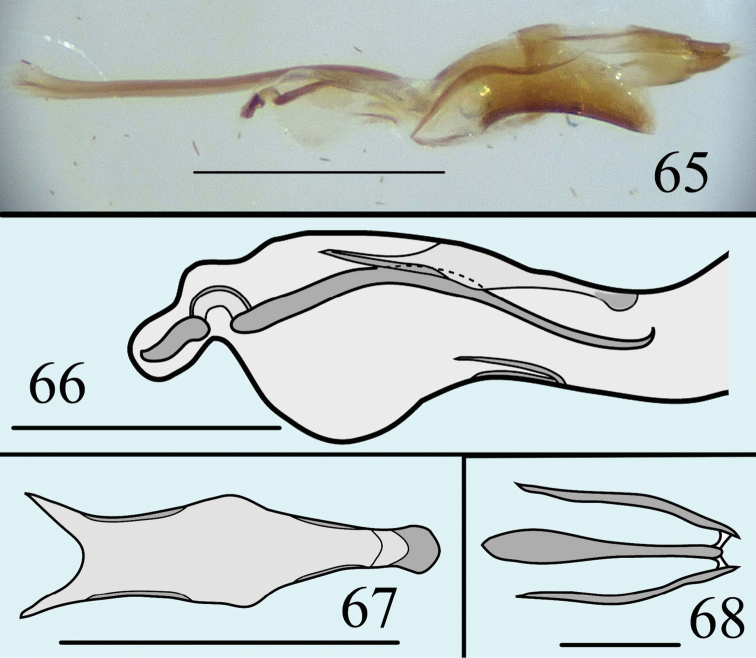
Male genitalia of *Neosternus higonius* in lateral view **65** Internal sac of *Neosternus higonius* in lateral view **66** Dorsal lobe of *Neosternus higonius* in dorsal view **6**, Ventral lobe of *Neosternus higonius* in dorsal view **68**. Scale=0.5mm (**65**), Scale=0.2mm (**66**, **67**), Scale=0.05mm (**68**).

**Figures 69–72.**
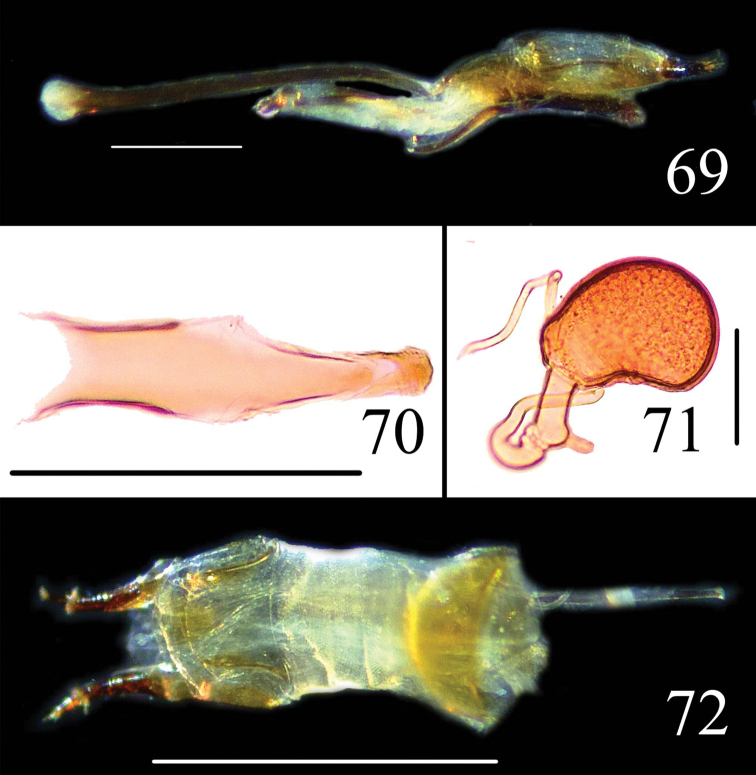
Male genitalia of *Neosternus hisamatsui* in lateral view **69** Dorsal lobe of *Neosternus hisamatsui* in dorsal view **70** Female genitalia of *Neosternus hisamatsui* in dorsal view **71** Female spermatheca of *Neosternus hisamatsui* **72**. Scale=1mm (**72**), Scale=0.3mm (**69**), Scale=0.2mm (**70**), Scale=0.1mm (**71**).

**Figures 73–75.**
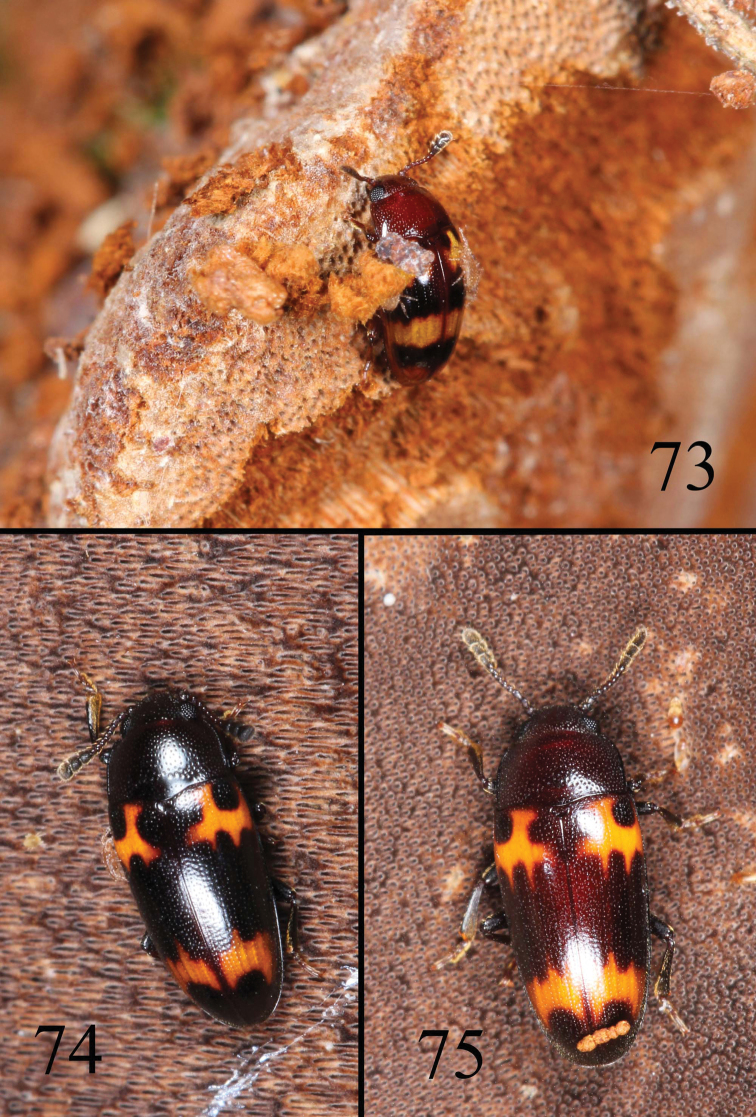
Habitat and adult feeding fungues of *Microsternus tricolor* **73** Habitat and adult feeding fungues of *Microsternus perforatus* **74–75**.

**Figures 76–78.**
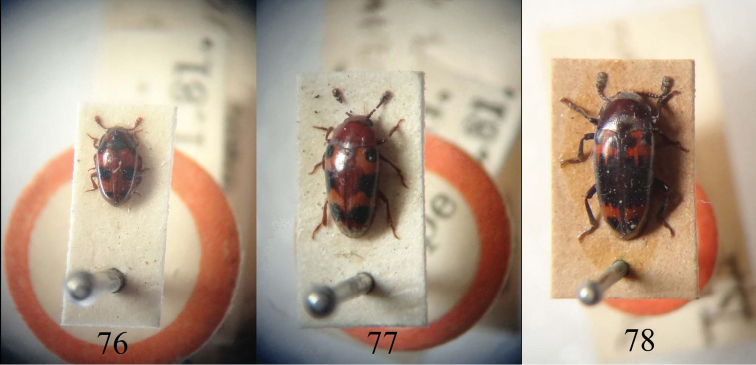
Type material of *Neosternus higonius* **76** Type material of *Microsternus tricolor* **77** Type material of *Microsternus perforatus* **78**. (NHM).

#### Distribution.

China, Japan.

#### Diagnosis.

This new genus can be distinguished from *Microsternus* by body elongate oval, head without the pit posterior to the postmandibular lobes, pronotum with a deep sulcus along each side, which is broadly margined in lateral view, male genitalia with flagellum bearing a arched mass on apical quarter (Figs 55–58). *Microsternus* body elongate, head with the pit posterior to the postmandibular lobes, pronotum without a deep sulcus along each side and narrowly margined in lateral view, male genitalia with flagellum bearing a straight mass on apical quarter (Figs 59–64).

#### Etymology.

As a uniquely new group within Dacnini, the generic name is derived from *Microsternus*, it is appropriate to call the genus “New *sternus*”, and to abbreviate and combine the roots into a single word. The gender is masculine.

#### Key to species of genus *Neosternus*

**Table:** 

1	Pronotum entirely reddish-brown, without markings	*Neosternushisamatsui* (Nakane)
–	Pronotum black or reddish-brown, with markings	2
2	Pronotum black, with a pair of red markings	*Neosternushigonius* (Lewis)
–	Pronotum reddish-brown, with a subtriangular black marking at each side of median part of anterior area and also a pair of transverse black markings at middle of base	*Neosternustaiwanus* (Chûjô)

### Neosternus higonius

(Lewis, 1887) comb. n.

http://species-id.net/wiki/Neosternus_higonius

Figs 34–35 , 38 , 43 , 48 , 53 , 55–56 , 65–68 , 76

Microsternus higonius Lewis, 1887.

#### Material examined.

**CHINA: Fujian Prov.:** 1♂, Wuyishan City, Guadun village, 27°44'N, 117°38'E, alt. 1200 m, 29.V.2012, Peng & Dai leg. (SNUC)

#### Description.

Body (Figs 34, 35, 76) elongate oval, length: 2.2–3.0 mm; width: 1.1–1.4 mm. Head and elytra reddish-brown; pronotum general black with reddish-brown sides; legs, palpi and base of antennae reddish-brown. Each elytron with three or four black bands.

Head width between eyes = 8 times eye diameter in dorsal view; punctation coarse, sparse, separated by 3-4 puncture diameters; epistome truncate, lacking marginal line on anterior margin; stridulatory files not evident. Eyes coarsely facetted. Antennomere III about 1.8 times as long as IV; antennomere VIII slightly wider than VII, about 1.2 times as wide as long; antennomere IX trapezoidal; antennomere X transverse; antennomere XI almost elliptic; relative lengths of antennomeres II–XI: 15: 18: 10: 10: 10: 10: 10: 14: 15: 17. Maxillary and labial terminal palpomeres acuminate, sensory area restricted to apex. Mentum broad with anterior projection, almost triangular, slightly more than 3.5 times wider than long.

Pronotum arched, widest at base (pl/pw = 0.55); narrowed from base to apex, with a deep sulcus along each side, which is broadly margined and the bordering gradually widened anteriorly (Fig. 48), which formed thicken lines in lateral view (Fig. 53); disk coarsely and sparsely punctured, except the impunctate medio-basal area, which is limited by an arched transverse row of coarse punctures.

Prosternum (Fig. 38) with median area including its process elevated in an elongate triangular plane, which is distinctly bordered by a ridge on both sides and shortly rounded-subtruncate in front, bearing a few fine punctures; sides rugose, coarsely and densely punctured. Mesosternum almost concealed by prosternal process, impunctate as the mesepisterna, which is somewhat concave. Metasternum rather sparsely and strongly punctured on lateral areas, some finer punctures on median area, with a pair of mesocoxal lines strongly divergent posteriorly. Abdomen rather strongly and closely punctured, but median areas of four basal visible sternites and medio-basal area of last visible sternite with few punctures respectively; without metacoxal lines on basal visible sternite. Legs rather robust.

Scutellum pentagonal, with each corner rounded, flattish and almost impunctate on surface.

Elytra strongly convex, with eight striae of distinct punctures on each elytron and each interstice with a row of extremely fine punctures.

Male genitalia (Fig. 65) with flagellum (Fig. 66) curved, bearing a dorsal, arched, cartilaginous mass on apical quarter; flagellar apex acute with a well-separated ventral process; dorsal lobe of internal sac with separated front and triangular end (Fig. 67); ventral lobe of internal sac trident-like (Fig. 68).

#### Distribution.

China, Japan.

#### Diagnosis.

Characterized by its small body and black pronotum.

### Neosternus hisamatsui

(Nakane, 1982) comb. n.

http://species-id.net/wiki/Neosternus_hisamatsui

Figs 36–37 , 39 , 44 , 49 , 54 , 57–58 , 69–72

Microsternus hisamatsui Nakane, 1982

#### Material examined.

**CHINA: CHINA: Guangxi Prov.:** 3♂♂, 2♀♀, Shangsi County, Mt. Shiwandashan, 21°54'N, 107°53'E, alt. 300–500 m, 25.IV.2011, PENG, ZHAI & ZHU leg. (SNUC); 1♂, Shangsi County, Mt. Shiwandashan, 21°54'N, 107°53'E, alt. 300–500 m, 4.V.2011, Liang Tang leg. (SNUC)

#### Description.

Body (Figs 36, 37) elongate oval, length: 2.4–3.0 mm; width: 1.2–1.4 mm. Head and elytra reddish-brown; pronotum general reddish-brown; legs, palpi and base of antennae reddish-brown. Each elytron with two to four black bands.

Head width between eyes = 8 times eye diameter in dorsal view; punctation coarse, sparse, separated by 3-4 puncture diameters; epistome truncate, lacking marginal line on anterior margin; stridulatory files not evident. Eyes coarsely facetted. Antennomere III about 1.8 times as long as IV; antennomere VIII slightly wider than VII, about 1.2 times as wide as long; antennomere IX trapezoidal; antennomere X transverse; antennomere XI almost elliptic; relative lengths of antennomeres II–XI: 15: 18: 10: 10: 10: 10: 10: 14: 15: 17. Maxillary and labial terminal palpomeres acuminate, sensory area restricted to apex. Mentum broad with anterior projection, almost triangular, slightly more than 3.5 times wider than long.

Pronotum arched, widest at base (pl/pw = 0.55); narrowed from base to apex, with a deep sulcus along each side, which is broadly margined and the bordering gradually widened anteriorly (Fig. 49), which formed thicken lines in lateral view (Fig. 54); disk coarsely and sparsely punctured, except the impunctate medio-basal area, which is limited by an arched transverse row of coarse punctures.

Prosternum (Fig. 39) with median area including its process elevated in an elongate triangular plane, which is distinctly bordered by a ridge on both sides and shortly rounded-subtruncate in front, bearing a few fine punctures; sides rugose, coarsely and densely punctured. Mesosternum almost conceled by prosternal process, impunctate as the mesepisterna, which is somewhat concave. Metasternum rather sparsely and strongly punctured on lateral areas, some finer punctures on median area, with a pair of mesocoxal lines strongly divergent posteriorly. Abdomen rather strongly and closely punctured, but median areas of four basal visible sternites and medio-basal area of last visible sternite with few punctures respectively; without metacoxal lines on basal visible sternite. Legs rather robust.

Scutellum pentagonal, with each corner rounded, flattish and almost impunctate on surface.

Elytra strongly convex, with eight striae of distinct punctures on each elytron and each interstice with a row of extremely fine punctures.

Male genitalia (Fig. 69) with flagellum (Figs 57–58) curved, bearing a dorsal, arched, cartilaginous mass on apical quarter; flagellar apex acute with a well-separated ventral process; dorsal lobe (Fig. 70) of internal sac with separated front and triangular end; ventral lobe of internal sac trident-like.

Female genitalia (Fig. 72) and spermatheca (Fig. 71) simple.

#### Distribution.

China, Japan.

#### Diagnosis.

Characterized by its small body and entirely reddish-brown pronotum.

#### Comment.

*Neosternus hisamatsui* Nakane is similar to *Neosternus higonius* Lewis in the form and color pattern of the body. *Neosternus hisamatsui* can be distinguished from *Neosternus higonius* by the pronotum entirely reddish-brown. *Neosternus higonius* has pronotum black with reddish-brown sides. Though the male genitalia and dorsal lobe of internal sac of *Neosternus higonius* is similar to *Neosternus hisamatsui*, with only one specimen of *Neosternus higonius* available and no Japanese specimens, we can’t consider *Neosternus hisamatsui* as a synonym of *Neosternus higonius*. This should be considered after maor materials are available for study.

### Neosternus taiwanus

(Chûjô, 1976) comb. n.

http://species-id.net/wiki/Neosternus_taiwanus

Microsternus taiwanus Chûjô, 1976.

#### Distribution.

China (Taiwan).

#### Diagnosis.

Characterized by its small body and markings of pronotum.

#### Comment.

Chûjô described *Microsternus taiwanus* from Taiwan. According Chûjô’s description, *Neosternus taiwanus* is very similar to *Neosternus higonius*. The only difference between these two species were the bands of pronotum and elytra as noted in the key to species. No specimens are available for study.

## Supplementary Material

XML Treatment for Microsternus

XML Treatment for Microsternus pengzhongi

XML Treatment for Microsternus perforatus

XML Treatment for Microsternus tricolor

XML Treatment for Neosternus

XML Treatment for Neosternus higonius

XML Treatment for Neosternus hisamatsui

XML Treatment for Neosternus taiwanus
